# Clonal expansion of community-associated meticillin-resistant Staphylococcus aureus (MRSA) in people who inject drugs (PWID): prevalence, risk factors and molecular epidemiology, Bristol, United Kingdom, 2012 to 2017

**DOI:** 10.2807/1560-7917.ES.2019.24.13.1800124

**Published:** 2019-03-28

**Authors:** Simon Packer, Bruno Pichon, Stephen Thompson, Jane Neale, Jacquelyn Njoroge, Rachel M Kwiatkowska, Isabel Oliver, Maggie Telfer, Michel Doumith, Camillus Buunaaisie, Ellen Heinsbroek, Noreen Hopewell-Kelly, Monica Desai, Vivian Hope, Owen Martin Williams, Angela Kearns, Mathew Hickman, Maya Gobin

**Affiliations:** 1 Field Epidemiology Service, Public Health England, Bristol, United Kingdom; 2 Antimicrobial Resistance and Healthcare Associated Infections Reference Unit, National Infection Service, Public Health England, London, United Kingdom; 3 Public health laboratory Bristol, Public Health England, Bristol, United Kingdom; 4 Bristol Drugs Project, Bristol, United Kingdom; 5 Blood Borne Virus Section, HIV & STI Department, National Infection Service, Public Health England, London, United Kingdom; 6 NIHR Health Protection Research Unit in Evaluation of Interventions at University of Bristol, Bristol, United Kingdom; 7 Infectious Diseases Research Department, King Abdullah International Medical Research Center, Riyadh, Saudi Arabia; 8 King Saud bin Abdulaziz University for Health Sciences, Riyadh, Saudi Arabia; 9 University of West England, Bristol, United Kingdom; 10 Public Health Institute, Liverpool John Moores University, Liverpool, United Kingdom; 11 School of Social and Community Medicine, University of Bristol, Bristol, United Kingdom; 12 Authors contributed equally to the work and share last authorship

**Keywords:** Meticillin-Resistant Staphylococcus aureus, Staphylococcal epidemiology, Staphylococcal Infections/transmission, MRSA, Sepsis, Injecting drug use, Intravenous, Substance Abuse, Intravenous: complications, Substance Abuse, Intravenous: microbiology, Drug users, Substance-Related Disorders/complications, Substance-Related Disorders/microbiology, Sequence Analysis, DNA, Whole-genome sequencing, United Kingdom, Community acquired infections, Community acquired infections epidemiology, Community acquired infections microbiology

## Abstract

Background: In 2015, Bristol (South West England) experienced a large increase in cases of meticillin-resistant *Staphylococcus aureus* (MRSA) infection in people who inject drugs (PWID).

Aim: We aimed to characterise and estimate the prevalence of MRSA colonisation among PWID in Bristol and test evidence of a clonal outbreak.

Methods: PWID recruited through an unlinked-anonymous community survey during 2016 completed behavioural questionnaires and were screened for MRSA. Univariable logistic regression examined associations with MRSA colonisation. Whole-genome sequencing used lineage-matched MRSA isolates, comparing PWID (screening and retrospective bacteraemia samples from 2012-2017) with non-PWID (Bristol screening) in Bristol and national reference laboratory database samples.

Results: The MRSA colonisation prevalence was 8.7% (13/149) and was associated with frequently injecting in public places (odds ratio (OR): 5.5; 95% confidence interval (CI):1.34–22.70), recent healthcare contact (OR: 4.3; 95% CI: 1.34–13.80) and injecting in groups of three or more (OR: 15.8; 95% CI: 2.51–99.28). People reporting any one of: injecting in public places, injection site skin and soft tissue infection or hospital contact accounted for 12/13 MRSA positive cases (sensitivity 92.3%; specificity 51.5%). Phylogenetic analysis identified a dominant clade associated with infection and colonisation among PWID in Bristol belonging to ST5-SCCmecIVg.

Conclusions: MRSA colonisation in Bristol PWID is substantially elevated compared with general population estimates and there is evidence of clonal expansion, community-based transmission and increased infection risk related to the colonising strain. Targeted interventions, including community screening and suppression therapy, education and basic infection control are needed to reduce MRSA infections in PWID.

## Introduction

Meticillin-resistant *Staphylococcus aureus* (MRSA) can exist as a harmless commensal or a potentially life-threatening pathogen [1,2]. Clinical presentations range from localised skin and soft tissue infections (SSTIs) to disseminated blood stream infections. These infections are responsible for substantial healthcare costs, morbidity and mortality [2-4]. The United Kingdom (UK) government have adopted a zero tolerance approach to avoidable healthcare associated infections with a focus on MRSA bacteraemia [5,6]. However, this approach is controversial as organisms can be introduced through multiple independent sources [5].

MRSA can survive in a range of ecological settings, interact with and colonise the human host and develop antimicrobial resistance via a range of mechanisms [4]. These traits allow MRSA to spread between populations and species exploiting niches and opening up footholds to establish reservoirs within different settings [4]. MRSA was initially thought to be confined to healthcare settings (HA-MRSA) but during the 1980s infections were noticed in the community (CA-MRSA) and in the early 2000s infections were also identified in humans associated with exposure to livestock (LA-MRSA) [1,7]. Colonising MRSA can be transmitted from person-to-person and introduced into the body when host defences are breached [1,8,9]. This is apparent in communities of people who inject drugs (PWID), with outbreaks previously reported in England and the United States (US) resulting in substantial morbidity and mortality [10-12]. Studies in Switzerland (2001), Canada (2006) and the US (2012) have found a high MRSA colonisation prevalence in PWID ranging from 5.7 to 18.6% [10,11,13]. These high prevalence estimates contrast sharply with general population estimates of < 0.1–1.5% and appear to be driven in part by frequent healthcare contact [10,13-18].

In the UK and Europe there is limited information on the incidence of infection and prevalence of colonisation associated with MRSA in PWID. It has been shown that symptoms of probable SSTI at injection sites are common, with 36% of PWID reporting these in a national survey in 2016 [19,20]. These reports, however, are not laboratory confirmed and do not provide information on the aetiological agent. Infections in PWID are often exacerbated through poor and/or delayed health-seeking behaviours, resulting in more serious and difficult to treat infections and substantial costs [21].

Bristol is a city in the South West of England with a population of 459,000; it is estimated that there are between 2,025–2,564 persons injecting drugs [22]. Bristol has the highest prevalence of crack use and sixth highest opiate use in England [23].

In the second half of 2014 the health community in Bristol identified an increase in the number MRSA bacteremias occurring in PWID. The increase was initially detected through the hospital post-infection review process. Following further investigation by the Field Epidemiology Service in Public Health England it was found that infections in PWID were a growing proportion of all MRSA cases reported in Bristol with the number more than doubling from 19 in 2013 to 45 in 2014, this increase was sustained in 2015 (46 infections) and up to August 2016 (37 infections). Infections in PWID were often serious with a considerable number resulting in protracted hospital admissions, amputations and/or death. Between January 2014 and August 2016, 18% of all reported infections in PWID were bloodstream infections (data not shown). This increase in MRSA among PWID in Bristol was in contrast to the national decline in MRSA bacteraemia rates from 2007 to 2017 [24]. Only MRSA bacteraemia samples were routinely sent for typing and there was no information on colonisation within the community. We therefore aimed to estimate the prevalence of MRSA among PWID in Bristol, explore the genetic relatedness of these samples compared with other PWID MRSA bacteraemia isolates and non-PWID isolates, identify injecting and non-injecting risk factors associated with infection and provide evidence to inform development and/or implementation of population specific control interventions. The prospective study was initiated from January 2016, with data collection occurring for 6 weeks from October 2016. Retrospective bacteraemia isolates in PWID occurring between 2012 to 2017 were examined.

## Methods

### Study population and setting

In 2016, a cross-sectional survey of PWID living in the City of Bristol was undertaken in partnership with the national Unlinked Anonymous Monitoring (UAM) survey of PWID [25]. The UAM has been running since 1990 and is an annual cross-sectional survey that recruits PWID from across England, Wales and Northern Ireland. The methodological details have been reported previously [26,27].

PWID in the City of Bristol were recruited through fixed site and mobile needle and syringe programme (NSP) using non-probability quota sampling [28]. The sample was purposely recruited to reflect age and sex data held by Bristol Drugs Project (BDP). BDP are a charity that provide drug and alcohol services within the City of Bristol.

### Data collection

Recruited participants completed an expanded version of the UAM questionnaire, which had been piloted on a group of BDP service users. Information on age and sex, homelessness, prior imprisonment, psychoactive drug use, uptake of health services and sexual behaviours was collected [29]. Questions were added according to a priori hypotheses relating to MRSA colonisation: living conditions (accommodation type, access to running water and living arrangements); injecting practice, such as injection site (arm, leg, groin, etc.), physical location (home, outside, squat, etc.), person-to-person contact (numbers of injecting companions); and previous infections (SSTI and MRSA). Data were double entered from paper questionnaires into a validated Epidata v3.1 data collection form [30]. Data inconsistencies were checked against original paper forms by the data entry team. Data were cleaned and recoded using R v3.2.0 [31].

### Data analysis

People were excluded from the analysis if they did not report injecting in the past year or if they had already completed the UAM survey that year. We identified factors associated with MRSA colonisation using univariable logistic regression and calculated odds ratios (OR) and corresponding 95% confidence intervals (CI).

We defined groups of people at greater risk of colonisation by examining combinations of risk factors chosen based upon the univariable analysis (OR > 2.5) and potential for targeting interventions. Factors representing recent MRSA colonisation were excluded. Risk factor combinations were assessed in terms of sensitivity, specificity, receiver operator curve (ROC) and positive predictive value (PPV). A ROC value of 0.70 or above was used as a threshold for inclusion [32].

#### Microbiological testing

Trained BDP staff members collected groin and nasal swabs from participants. Swabs were cultured onto Brilliance Staph 24 agar (Oxoid). Presumptive *S.aureus* were initially identified using matrix-assisted laser desorption ionization-time of flight mass spectrometry MALDI-TOF (Bruker Daltonik GmbH, Germany) and MRSA were identified by antibiotic susceptibility testing using VITEK 2 (software v07.01 and card name AST-P635, bioMérieux). Colonised participants were defined as people living in the City of Bristol who reported injecting within the last past year and found to be positive for MRSA colonisation in nasal and/or groin sites.

#### Whole-genome sequence analysis and phylogenetic analysis

All MRSA were subjected to WGS, bioinformatic and phylogenetic analysis as described previously, with N315 (NC002745) being used as reference [33]. Briefly, genomic DNA was extracted using the QIAsymphony platform (Qiagen, Hilden, Germany), fragmented and tagged for multiplexing with Nextera XT DNA Sample Preparation Kits, followed by paired-end sequencing on an Illumina HiSeq 2500 platform to produce 100 bp paired-end reads (Illumina, Cambridge, UK) and a coverage above 30x [33]. For phylogenetic analysis, sequence reads were mapped to the N315 reference strain (NC002745) using BWA(0.7.5). Single Nt Polymorphisms (SNPs) were called using GATK2.6.5. Genetic relatedness was determined using only high quality SNPs (AD genotype = 0.9). Coverage was above 95% of the reference genome. SNPs were concatenated and aligned allowing 20% of Ns and gaps. Clusters were defined by hierarchical clustering using single linkage and SNP threshold of 150 using fastcluster in R (Supplementary Table S1) [34]. Phylogeny was inferred from concatenated SNP alignment by using RaxML (Maximum Likelihood using GTR substitution model and 100 bootstrap) [35]. The tree was visualised using interactive Tree Of Life (iTOL), pairwise SNP distance matrix was calculated excluding Ns and gaps (Supplementary Table S2).

Bristol PWID colonisation isolates identified as belonging to a dominant MRSA clone were compared with lineage-matched isolates that had been subjected to WGS in the Antimicrobial Resistance and Healthcare Associated Infections Reference Unit in Public Health England (PHE), specifically: (i) retrospective Bristol PWID MRSA bacteraemia isolates, (ii) non-PWID MRSA carriage isolates from pre-admission screening swabs from the University Hospital Bristol (UHB) and (iii) contemporaneous representative MRSA from the PHE national reference laboratory archive. Susceptibility data were not available for these comparator isolates. The retrospective Bristol PWID bacteraemia isolates were identified through record linkage between drug services data and laboratory reports of all samples processed at UHB laboratory from 2012 to 2016. Repeat, non-duplicate, infections were included and defined as any MRSA with a sample date greater than 14 days apart. Non-PWID UHB admission screening samples were selected from a convenience sample of MRSA positive UHB admission screening samples collected during October 2016. This time period was contemporaneous with the PWID swabbing element of the study. Details of the UHB screening criteria have been described previously [36]. These were checked against hospital records to ensure they were from people not reporting injecting drug use. PHE national reference laboratory archive isolates had information on the geographical location and presence of injecting risk factors collated.

Descriptive statistics (mean, median, minimum, maximum and interquartile range), frequencies and percentages were used to compare epidemiological characteristics of people within and between clusters.

### Ethical statement

Participants provided verbal consent when enrolled into the study.

The study received ethical approval from the London research ethics committee (REC reference: 98/2/051).

## Results

### Study population

There were 153 survey participants of which 149 reported injecting in the past year (2015 to 2016) and were included in the analysis. The majority of participants were male (84%; 128/153) and were aged 35–44 years (46%; 71/153). The median age was similar for men (39, Interquartile range (IQR): 34.5–46) and women (40; IQR: 31–45). The majority (95%; 142/149) reported injecting in the past month, commonly with opioids (35%; 50/142) or opioids and stimulant combinations (30%; 42/142). Participants typically reported injecting into their arms (56%; 79/142) and/or groin (52%; 74/142); 44% (65/149) reported homelessness within the past year. Over a third (37%; 47/142) of people self-reported symptoms of a previous SSTI at their injection site in the past year.

Of the participating PWID, 13 were colonised with MRSA, giving a prevalence of 8.7%. Nineteen of the 149 PWID reported a previous SSTI due to MRSA; 13 (10%; 13/136) in non-colonised and six (6/13) in colonised PWID . Four of these occurred in the past 3 months and all reported being prescribed decolonisation therapy (nasal cream and body wash) in the past month.

Of the 136 non-colonised participating PWID, 17 (13%) reported a previous MRSA infection, five of them within the past 3 months. Eleven of the 17 people reporting a previous infection reported a SSTI and four reported previous decolonisation.

### Factors associated with MRSA colonisation

We identified several factors strongly associated with MRSA colonisation among PWID in Bristol. Participants who reported most frequently injecting in public places (OR: 5.5; 95% CI: 1.34–22.70), hospital contact in the past month (OR: 4.3; 95% CI: 1.34–13.80), most frequently injecting in a group of three or more (OR: 15.8; 95% CI: 2.51–99.28) and experiencing an MRSA infection in the past 3 months (OR: 13.6; 95% CI: 2.98–62.17) were associated with MRSA colonisation. Weaker associations were identified for SSTI at an injecting site in past year (OR: 2.8; 95% CI: 0.89–8.85), homelessness in the past year (OR: 3.2; 95% CI: 0.94–10.96), groin injecting (OR: 3.8; 95% CI: 0.99–14.23) and previous deep vein thrombosis (DVT) comorbidity (OR: 2.6; 95% CI: 0.81–8.18) (Table 1).

**Table 1:** Univariable analysis of factors associated with meticillin-resistant *Staphylococcus aureus* in colonisation, Bristol, 2016 (n = 149)

Variable	Value	Neg	%	Pos	%	Total	OR	95% CI
Skin and soft tissue infection in the past year	No	96	94.1	6	5.9	102	Ref	NA
	Yes	40	85.1	7	14.9	47	2.8	0.89–8.85
Most frequently injecting location	House own/friend	75	94.9	4	5.1	79	Ref	NA
	Hostel, squat, other	44	91.7	4	8.3	48	1.7	0.41–7.16
	Public places	17	77.3	5	22.7	22	5.5	1.34–22.73
Hospital contact past month	No	107	94.7	6	5.3	113	Ref	NA
	Yes	29	80.6	7	19.4	36	4.3	1.34–13.8
Homeless past year	No	80	95.2	4	4.8	84	Ref	NA
	Yes	56	86.2	9	13.8	65	3.2	0.94–10.96
Groin inject in the past month	No	72	96.0	3	4.0	75	Ref	NA
	Yes	64	86.5	10	13.5	74	3.8	0.99–14.23
Ever experienced a DVT co-morbidity	No	102	93.6	7	6.4	109	Ref	NA
	Yes	34	85.0	6	15.0	40	2.6	0.81–8.18
Frequently inject in groups	Own	79	94.0	5	6.0	84	Ref	NA
	Less than three people	54	91.5	5	8.5	59	1.5	0.4–5.3
	Three or more people	3	50.0	3	50.0	6	15.8	2.51–99.28
Previous MRSA Infection	No previous infection	119	94.4	7	5.6	126	Ref	NA
	> 3 months ago	12	85.7	2	14.3	14	2.8	0.53–15.2
	≤ 3 months ago	5	55.6	4	44.4	9	13.6	2.98–62.17

CI: confidence interval; DVT: deep vein thrombosis; MRSA: meticillin-resistant *Staphylococcus aureus*; NA: not applicable; Neg: negative for MRSA colonisation; OR: odds ratio; Pos: positive for MRSA colonisation; Ref: reference.

Six indicators met our criteria for grouping: injecting in public places, hospital contact, injecting in a group of three or more people, SSTI, homelessness in the past year and groin injecting. We identified four groups according to different permutations of 4 of 6 indicator variables, which defined Bristol PWID with greater odds of MRSA colonisation with adequate sensitivity and specificity (ROC 0.7). Group 1 defined people who reported frequently injecting in public places or SSTI in past year or healthcare contact in past month. Group 2 people included people reporting injected in a group of three or more or frequently inject in public places or SSTI in past year or healthcare contact in past month. Group 3 included people with a SSTI in past year or healthcare contact in past month. Group 4 included people reporting injecting in a group of three or more or SSTI in past year or healthcare contact in past month. Group 1 and 2 best explained MRSA colonisation accounting for 12 of 13 colonised participants with high sensitivity (> 92%) and moderate specificity (≥ 50%) (Table 2).

**Table 2:** Univariable analysis of the association between clinical assessment group classifications and MRSA colonisation, sensitivity, specificity and positive predictive value in sampled PWID, 2016

Group	Value	Neg	Pos	OR	95% CI	P value	Sensitivity (%)	Specificity (%)	ROC	PPV (%)
Group 1	No	70	1	Ref	NA	NA	NA	NA	NA	NA
	Yes	66	12	12.57	1.77–551.65	0.004	92.3	51.5	0.72	15.4
Group 2	No	68	1	Ref	NA	NA	NA	NA	NA	NA
	Yes	68	12	11.86	1.67–520.34	0.005	92.3	50.0	0.71	15.0
Group 3	No	78	2	Ref	NA	NA	NA	NA	NA	NA
	Yes	58	11	7.31	1.51–70.36	0.008	84.6	57.4	0.71	15.9
Group 4	No	75	2	Ref	NA	NA	NA	NA	NA	NA
	Yes	61	11	6.69	1.38–64.35	0.011	84.6	55.1	0.70	15.3
Total		136	13	NA	NA	NA	NA	NA	NA	NA

CI: confidence interval; MRSA: meticillin-resistant *Staphylococcus aureus*; NA: not applicable; Neg: negative; OR: odds ratio; Pos: positive; PPV: positive predictive value; PWID: people who inject drugs; Ref: reference; ROC: receiver operator curve; SSTI: Skin and soft tissue infections.

Group 1 defined people who reported frequently injecting in public places or SSTI in past year or healthcare contact in past month. Group 2 people included people reporting injecting in a group of three or more or frequently injecting in public places or SSTI in past year or healthcare contact in past month. Group 3 included people with a SSTI in past year or healthcare contact in past month. Group 4 included people reporting injecting in a group of three or more or SSTI in past year or healthcare contact in past month.

### Microbiological analysis

In total, there were 16 Bristol PWID colonisation samples from our survey, 39 retrospective Bristol PWID bacteraemia samples and 25 non-PWID UHB admission screening samples. The 16 Bristol PWID colonisation isolates were recovered from 13 survey participants and included two phenotypically distinct isolates from one participant and two who were positive at both nose and groin sites. Genomic analysis showed the majority of the Bristol PWID colonisation MRSA (12/16) belonged to multilocus sequence type 5 (ST5), encoded staphylococcal cassette chromosome *mec* type IVg (SCC*mec*IVg) and were PVL-negative. The remainder belonged to ST1-IV (n = 3) or ST3919-IV (n = 1; a single locus variant of ST8). Greater heterogeneity was apparent among the non-PWID UHB admission screening samples with eight of 25 belonging to multilocus sequence type clonal complex 5 (CC5); the remainder comprised CC22 (n = 8), CC30 (n = 4), CC1 (n = 3), CC8 (n = 1) and CC59 (n = 1).

A phylogenetic tree of 71 ST5 MRSA (24 bacteraemia and 12 carriage isolates from PWID in Bristol, eight pre-admission screening swabs from UHB patients in Bristol and 27 from PHE national reference laboratory archives) is shown in Figure. The majority (68%; 48/71) belonged to a single lineage (ST5-SCC*mec*IVg) herein dubbed the ‘Bristol clade’. The ST5-IVg lineage has been infrequently noted it in hospitalised patients. Furthermore, the majority of bacteraemia cases observed in PWID are community onset (occurring < 48h following admission to hospital).

**Figure:**
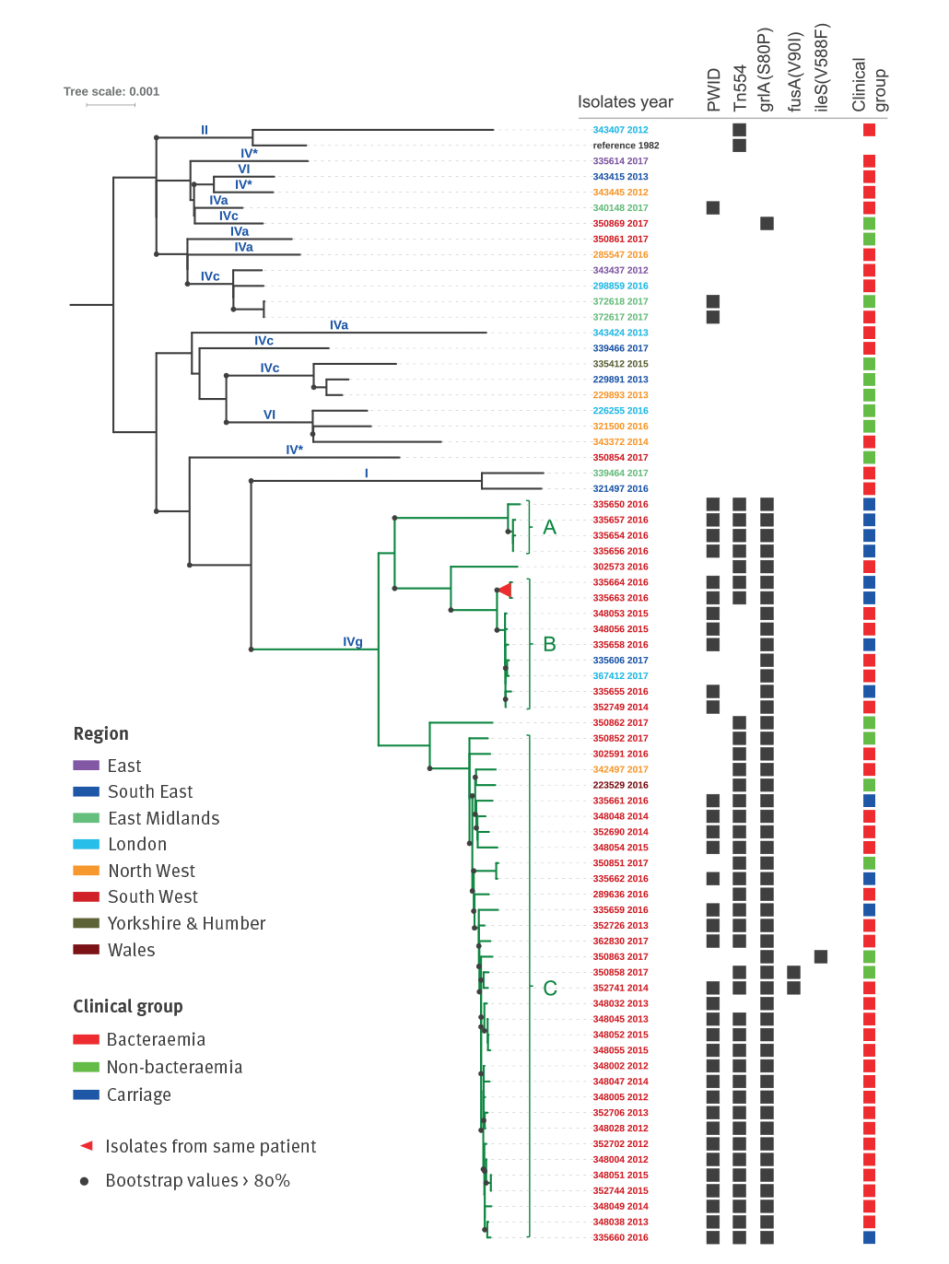
Maximum-likelihood phylogenetic tree based on SNPs in the core genome of 71 ST5-MRSA

SNPs: single nucleotide polymorphisms; ST5-MRSA: meticillin-resistant *Staphylococcus aureus* Sequence Type (ST) 5; PWID: People who inject drugs.

The tree was visualised using interactive Tree Of Life (iTOL); pairwise SNP distance matrix was calculated excluding Ns and gaps.

Maximum-likelihood phylogenetic tree based on SNPs in the core genome of 71 ST5-MRSA including: 24 bacteraemia and 12 carriage isolates from PWID in Bristol, eight carriage (pre-admission screening) isolates from patients in Bristol and 27 temporally related, geographically dispersed isolates from PHE national reference laboratory archives; reference genome N315 (NC002745).

SCCmectypes are denoted on the branches. Black squares indicate PWID status and presence of genetic markers associated with particular resistance traits. Isolate labels are coloured according to geographic region. Clinical groups comprised bacteraemia infection (n = 44), non-bacteraemia infection (n = 12) and carriage (n = 15). A, B, C designation denotes sub-clusters within Bristol clade at 150 SNP threshold. Scale of branch distance represents ca 140 SNPs.

Within the Bristol clade, three sub-clades were apparent. Sub-clade A comprised four PWID carriage isolates recovered in 2016. There were nine isolates in sub-clade B, recovered over a 4-year period (2014–17), comprising four carriage and three bacteraemia isolates from PWID in Bristol and two bacteraemia cases with injecting status unknown: one each from London and the South East of England. Sub-clade C was the largest and included 33 isolates recovered over 6-year period (2012–17); 31 were from Bristol (25 PWID, the injecting status for the other six was unknown) and included 23 bacteraemia, four carriage and four non-invasive samples. The remaining two included one each from non-PWID in Wales and North West England. The remaining 23 isolates (namely, nationally representative CC5 comparator isolates including three from PWID in the East Midlands and three from non-PWID UHB admission screening samples) in the tree recovered over the same timescale (2012–17) were phylogenetically heterogeneous; none of the Bristol PWID isolates were represented in this group. All were ST5 but none encoded SCC*mec*IVg, although multiple other SCC*mec* types were apparent and, in contrast to the Bristol clade, few resistance traits were identified (Figure). The PWID carriage samples that were part of the Bristol clade. Persons with isolates from different sub-clades varied in terms of their epidemiological metadata (Table 3).

**Table 3:** A description of the epidemiological metadata for PWID colonised with MRSA from the Bristol clade, by sub-clade, 2016

Characteristic	MRSA Bristol clade			
	Sub-clade A	Sub-clade B	Sub-clade C	Total
Hospital contact past month	3	3	0	6
Groin inject in the past month	2	3	3	8
Homeless past year	3	2	3	8
Infection at injection site	3	1	2	6
Previous MRSA infection in past 3 months	1	2	0	3
Most frequently injecting location in public places	2	1	1	4
Frequently inject in groups of three or more	2	1	0	3
Total	4	3	4	11

MRSA: meticillin-resistant *Staphylococcus aureus*; PWID: people who inject drugs.

In addition to SCC*mec*IVg, other resistance elements were also highly conserved among the Bristol clade isolates; specifically, Tn554 which encodes *ermA* and *ant(9)-Ia* (conferring resistance to erythromycin and spectinomycin respectively) and mutational resistance to fluoroquinolones (*grlA* S80F). Loss of Tn554 was apparent in seven sub-clade B isolates. Independent loss of this mobile element was observed in two sub-clade C isolates; a further two displayed mutational resistance to fusidic acid in an apparent single event (*fusA* V90I). One isolate carrying mutational resistance (*ileS* V588F) exhibited intermediate resistance to mupirocin on VITEK.

## Discussion

We combined WGS and epidemiological data to provide novel insights into an increase in MRSA among PWID in Bristol. This study was instigated due to a large increase in the number of MRSA infections in PWID in 2014. The MRSA colonisation prevalence among PWID in Bristol was around six times higher than the general population (8.7% vs up to 1.5%) but is broadly in line with previous studies of MRSA colonisation among PWID (5.7–18.6%) [10,13,14,16]. This puts Bristol PWID at increased risk due to the well-defined association between colonisation and infection [8]. The factors associated with MRSA colonisation were PWID who reported injecting in public places, recent healthcare contact and injecting in groups of three or more and reporting a SSTI.

The collective data indicate the establishment of a successful clade of CA-MRSA (dubbed the ‘Bristol clade’) associated with colonisation and infection among Bristol’s PWID population. The data suggest that there has been ongoing circulation and transmission within the PWID community over several years. More specifically, hierarchical clustering and phylogenetic analyses showed evidence of clonal expansion of an ST5-MRSA-IVg clade among PWID in Bristol between 2012 and 2017 indicating an association with this genotype and PWID risk group. This contrasts with the genetic heterogeneity observed among the non-PWID UHB admission screening samples which belonged to six different MLST-CCs. In addition, the Bristol clade is distinct from the dominant HA-MRSA strain circulating in the UK (CC22-SCC*mec*IVh; EMRSA-15) and the epidemiological and genomic data identify it as a PVL-negative community-associated type of MRSA [9]. Given our knowledge of MRSA epidemiology in hospitals in England (currently dominated by CC22-IVh and CC5-IVc), we regard the ST5-IVg clone identified among PWID as being a community-like MRSA because we have rarely noted it in hospitalised patients and the bacteraemia cases observed in PWID are community onset (occurring < 48h following admission to hospital). As has been noted for other MRSA lineages and ecological niches, we hypothesise that representatives of this clade have evolved and increased in fitness through adaptation to particular settings and populations [37]. Representatives of the Bristol clade were identified in 11 individuals where there was no evidence of injecting drug use (including seven pre-admission screening swabs from Bristol and four clinical infections occurring in geographically distinct regions in England and Wales). Additional information on these persons was not available, however other risk factors such as contact with PWID, homelessness or alcohol abuse may account for some/all of these cases. This is supported by evidence that networks of PWID can operate as reservoirs of infection with significant links to the general population [38]. This may provide evidence that this lineage is infiltrating wider population networks. An alternative explanation could be that a rare strain of MRSA from the general population has entered and spread through the Bristol PWID population. The study is unable to provide a definitive answer as to the source of this strain although we show clearly that MRSA is being spread between PWID and the general population.

The data indicate an association between the presence of a specific lineage of MRSA among PWID and developing an invasive infection. This is supported by the smaller number or absence of invasive samples belonging to other lineages. This association could be attributable to adaptation or tropism within this MRSA strain or the epidemiological characteristics of the affected groups, such as injecting practices [37].

A major limitation of this study is the sample size as it provided insufficient power to perform multivariable analysis and some of the associations could be subject to confounding. This issue was anticipated and the study was designed to provide a baseline from which further work could be conducted. Moreover, as there is no sampling frame for this population, we used a non-random sampling method to recruit participants. To mitigate this issue, we used a quota-based approach to ensure the sample was representative of the known of PWID population in Bristol. The age and sex distribution of our sample was similar to the PWID population engaged with the extensive NSP in Bristol, that involves both fixed and mobile programmes, as previously measured by BDP in 2015 (data not shown). The cross sectional design was not able to estimate incidence or rule out reverse causation between colonisation and risk factors. Finally, PHE reference laboratory holds data on PWID status however, this is poorly completed which could result in misclassification and under-estimate PWID exposure in all samples.

Taken together, the high colonisation prevalence, establishment of a successful clone of CA-MRSA within the PWID population, possible dissemination to the general population and the high number of invasive infections within a specific vulnerable group, we believe there is sufficient evidence for public health action. Currently we are not aware of any specific guidance in Europe or worldwide for the management of MRSA in PWID. Previous outbreaks have targeted enhanced wound care and basic hand hygiene interventions alongside improved access to healthcare; although the effectiveness of these measures remains untested [39]. Ideally, any intervention should be aimed towards targeting not only MRSA but bacterial infections in general. A more holistic approach is desirable particularly in light of outbreaks of invasive group A streptococci (iGAS) infections affecting PWID populations in the UK (2016 and 2017) and in Canada (2008). These were caused by the emergence of unusual strain types resulting in a substantial number of cases and could not be traced to a source [40-42]. A general approach is also likely to be more effective than suppression therapy on its own as it is widely recognised that MRSA decolonisation therapy (nasal cream and body wash) can be ineffective [43]. Apparent failure of eradication can be multi-factorial and, from our data, we do not know what treatment regimen was used and whether it was adhered to or not.

More broadly, a range of harm reduction measures have been shown to effectively reduce the risk of bacterial infections among PWID, such as provision of advice and education in good hygienic practices and basic infection prevention control [44]. Providing training in safe injection techniques, including cleaning of the injection site, can also lower bacterial infection risk [45,46]. There remains the need for upstream interventions, such as providing harm reduction resources, supervised injecting facilities and opioid substitution therapy, as these are excellent methods to reduce the overall number of infections among PWID [44,47-50]. The results of this study have been used to improve the post-infection review process and develop a pilot with planned evaluation for universal supply for of Chlorhexidine wipes to PWID through NSP. The findings from this study provide information to help inform the development of targeted interventions such as community-based screening, health promotion messaging, wound care, skin cleaning advice and suppression therapy with personal and environmental decontamination (washing clothes and bedding) [39,51].

From a wider perspective, molecular epidemiological initiatives locally, nationally and internationally should be encouraged to further our understanding of clonal shifts in MRSA not only within at risk groups such as PWID, but across all healthcare sectors. Such studies should be prospective in nature and utilise a social network approach to identify high-risk communities and factors associated with MRSA infection. There is a need to develop and evaluate the feasibility of community- and hospital-based interventions to prevent MRSA in PWID. Current issues centre on the complexity of managing PWID as inpatients, adherence to treatment and re-acquisition of MRSA within the community. These groups have frequent hospital contact, which could negatively impact on local infection control for MRSA; therefore, increasing awareness of local medical staff is vital to promote screening and the appropriate prescribing of suppression treatment to MRSA-positive PWID as is widely recommended on admission to hospital.

In conclusion, this study details the emergence of a CA-MRSA clone within Bristol’s PWID population that is circulating within the community and is responsible for a considerable number of invasive infections in PWID. Surveillance and further research are required locally, nationally and internationally to examine the epidemiology of this clone and identify areas/people at risk. Public health action is required to mitigate this on-going risk and protect PWID from MRSA and other bacterial infections.

## References

[1] Gordon RJ Lowy FD Pathogenesis of methicillin-resistant Staphylococcus aureus infection. Clin Infect Dis. 2008;46(S5) Suppl 5;S350-9. 10.1086/533591 18462090 PMC2474459

[2] Thomer L Schneewind O Missiakas D Pathogenesis of Staphylococcus aureus Bloodstream Infections. Annu Rev Pathol. 2016;11(1):343-64. 10.1146/annurev-pathol-012615-044351 26925499 PMC5068359

[3] Zetola N Francis JS Nuermberger EL Bishai WR Community-acquired meticillin-resistant Staphylococcus aureus: an emerging threat. Lancet Infect Dis. 2005;5(5):275-86. 10.1016/S1473-3099(05)70112-2 15854883

[4] Bal AM Coombs GW Holden MTG Lindsay JA Nimmo GR Tattevin P Genomic insights into the emergence and spread of international clones of healthcare-, community- and livestock-associated meticillin-resistant Staphylococcus aureus: Blurring of the traditional definitions. J Glob Antimicrob Resist. 2016;6:95-101. 10.1016/j.jgar.2016.04.004 27530849

[5] Török ME Harris SR Cartwright EJ Raven KE Brown NM Allison ME Zero tolerance for healthcare-associated MRSA bacteraemia: is it realistic? J Antimicrob Chemother. 2014;69(8):2238-45. 10.1093/jac/dku128 24788657 PMC4100711

[6] Department of Health and Social Care (DHSC). Technical Guidance for the 2012/13 Operating Framework. London: DHSC; 2011. Available from: https://assets.publishing.service.gov.uk/government/uploads/system/uploads/attachment_data/file/216413/dh_132045.pdf

[7] Price LB Stegger M Hasman H Aziz M Larsen J Andersen PS Staphylococcus aureus CC398: host adaptation and emergence of methicillin resistance in livestock. MBio. 2012;3(1):1-6. 10.1128/mBio.00305-11 22354957 PMC3280451

[8] Wertheim HF Melles DC Vos MC van Leeuwen W van Belkum A Verbrugh HA The role of nasal carriage in Staphylococcus aureus infections. Lancet Infect Dis. 2005;5(12):751-62. 10.1016/S1473-3099(05)70295-4 16310147

[9] Nimmo GR Coombs GW Community-associated methicillin-resistant Staphylococcus aureus (MRSA) in Australia. Int J Antimicrob Agents. 2008;31(5):401-10. 10.1016/j.ijantimicag.2007.08.011 18342492

[10] Leung NS Padgett P Robinson DA Brown EL Prevalence and behavioural risk factors of Staphylococcus aureus nasal colonization in community-based injection drug users. Epidemiol Infect. 2015;143(11):2430-9. 10.1017/S0950268814003227 25434806 PMC9150943

[11] Gilbert M MacDonald J Gregson D Siushansian J Zhang K Elsayed S Outbreak in Alberta of community-acquired (USA300) methicillin-resistant Staphylococcus aureus in people with a history of drug use, homelessness or incarceration. CMAJ. 2006;175(2):149-54. 10.1503/cmaj.051565 16804118 PMC1490001

[12] Otter JA French GL Community-associated meticillin-resistant Staphylococcus aureus in injecting drug users and the homeless in south London. J Hosp Infect. 2008;69(2):198-200. 10.1016/j.jhin.2008.02.014 18387697

[13] Fleisch F Zbinden R Vanoli C Ruef C Epidemic spread of a single clone of methicillin-resistant Staphylococcus aureus among injection drug users in Zurich, Switzerland. Clin Infect Dis. 2001;32(4):581-6. 10.1086/318716 11181121

[14] Al-Rawahi GN Schreader AG Porter SD Roscoe DL Gustafson R Bryce EA Methicillin-resistant Staphylococcus aureus nasal carriage among injection drug users: six years later. J Clin Microbiol. 2008;46(2):477-9. 10.1128/JCM.01596-07 18039800 PMC2238082

[15] Harbarth S François P Shrenzel J Fankhauser-Rodriguez C Hugonnet S Koessler T Community-associated methicillin-resistant Staphylococcus aureus, Switzerland. Emerg Infect Dis. 2005;11(6):962-5. 10.3201/eid1106.041308 15963298 PMC3367580

[16] Abudu L Blair I Fraise A Cheng KK Methicillin-resistant Staphylococcus aureus (MRSA): a community-based prevalence survey. Epidemiol Infect. 2001;126(3):351-6. 10.1017/S0950268801005416 11467791 PMC2869702

[17] Zanelli G Sansoni A Zanchi A Cresti S Pollini S Rossolini GM Staphylococcus aureus nasal carriage in the community: a survey from central Italy. Epidemiol Infect. 2002;129(2):417-20. 10.1017/S0950268802007434 12403117 PMC2869900

[18] Maudsley J Stone SP Kibbler CC Iliffe SR Conaty SJ Cookson BD The community prevalence of methicillin-resistant Staphylococcus aureus (MRSA) in older people living in their own homes: implications for treatment, screening and surveillance in the UK. J Hosp Infect. 2004;57(3):258-62. 10.1016/j.jhin.2004.03.023 15236857

[19] Hope V Kimber J Vickerman P Hickman M Ncube F Frequency, factors and costs associated with injection site infections: findings from a national multi-site survey of injecting drug users in England. BMC Infect Dis. 2008;8(1):120. 10.1186/1471-2334-8-120 18801177 PMC2600824

[20] Public Health England (PHE). Data tables of the Unlinked Anonymous Monitoring Survey of HIV and Hepatitis in People Who Inject Drugs. London: PHE; 2017. Available from: https://assets.publishing.service.gov.uk/government/uploads/system/uploads/attachment_data/file/633204/UAM_Survey_of_PWID_data_tables_2017.pdf

[21] Hope VD Ncube F Parry JV Hickman M Healthcare seeking and hospital admissions by people who inject drugs in response to symptoms of injection site infections or injuries in three urban areas of England. Epidemiol Infect. 2015;143(1):120-31. 10.1017/S0950268814000284 24568684 PMC9206814

[22] Ward Z Platt L Sweeney S Hope VD Maher L Hutchinson S Impact of current and scaled-up levels of hepatitis C prevention and treatment interventions for people who inject drugs in three UK settings-what is required to achieve the WHO’s HCV elimination targets? Addiction. 2018;113(9):1727-38. 10.1111/add.14217 29774607 PMC6175066

[23] Public Health England (PHE). Opiate and crack cocaine use: prevalence estimates by local area. Estimates of the number of opiate and crack cocaine users in local areas from 2014 to 2015. [Accessed 21 Mar 2019]. Available from: https://www.gov.uk/government/publications/opiate-and-crack-cocaine-use-prevalence-estimates-for-local-populations.

[24] Public Health England (PHE). Annual Epidemiological Commentary: Mandatory MRSA, MSSA and E. coli bacteraemia and C. difficile infection data. London: PHE; 2017. Available from: https://webarchive.nationalarchives.gov.uk/20180410202808/https://www.gov.uk/government/statistics/mrsa-mssa-and-e-coli-bacteraemia-and-c-difficile-infection-annual-epidemiological-commentary

[25] Public Health England (PHE). Unlinked anonymous HIV and viral hepatitis monitoring among PWID: 2016 report. London: PHE; 2016. Available from: https://assets.publishing.service.gov.uk/government/uploads/system/uploads/attachment_data/file/538321/hpr2316_uampwid.pdf

[26] Noone A Durante AJ Brady AR Majid F Swan AV Parry JV HIV infection in injecting drug users attending centres in England and Wales, 1990-1991. AIDS. 1993;7(11):1501-7. 10.1097/00002030-199311000-00015 8280418

[27] Hope VD Judd A Hickman M Sutton A Stimson GV Parry JV HIV prevalence among injecting drug users in England and Wales 1990 to 2003: evidence for increased transmission in recent years. AIDS. 2005;19(11):1207-14. 10.1097/01.aids.0000176222.71355.a1 15990575

[28] Cumming RG Is probability sampling always better? A comparison of results from a quota and a probability sample survey. Community Health Stud. 1990;14(2):132-7. 10.1111/j.1753-6405.1990.tb00033.x 2208977

[29] Public Health England (PHE). Unlinked anonymous HIV and viral hepatitis monitoring among PWID: 2017 report. London: PHE; 2017. Available from: https://assets.publishing.service.gov.uk/government/uploads/system/uploads/attachment_data/file/633688/hpr2617_uam-pwid.pdf

[30] Christiansen TB, Lauritsen JM, editors. EpiData - Comprehensive Data Management and Basic Statistical Analysis System. Odense: EpiData Association; 2010. Available from: https://www.epidata.dk/credit.htm

[31] R Core Team. (2017) R: A language and environment for statistical computing. Available from: https://www.r-project.org/

[32] Bewick V Cheek L Ball J Statistics review 13: receiver operating characteristic curves. Crit Care. 2004;8(6):508-12. 10.1186/cc3000 15566624 PMC1065080

[33] Lahuerta-Marin A Guelbenzu-Gonzalo M Pichon B Allen A Doumith M Lavery JF First report of lukM-positive livestock-associated methicillin-resistant Staphylococcus aureus CC30 from fattening pigs in Northern Ireland. Vet Microbiol. 2016;182:131-4. 10.1016/j.vetmic.2015.11.019 26711039

[34] Journal of statistical Software. Daniel Müllner. Fastcluster: Fast Hierarchical, Agglomerative Clustering Routines for R and Python. Published online 2013. Available from: https://www.jstatsoft.org/article/view/v053i09

[35] Stamatakis A RAxML version 8: a tool for phylogenetic analysis and post-analysis of large phylogenies. Bioinformatics. 2014;30(9):1312-3. 10.1093/bioinformatics/btu033 24451623 PMC3998144

[36] University Hospitals Bristol, National Health Service (NHS) Foundation Trust. MRSA screening Policy and protocol. Bristol: NHS; 2009. Available from: http://www.uhbristol.nhs.uk/files/nhs-ubht/MRSA%20Screening%20UH%20Bristol%20(V01,%20Mar%2009).pdf

[37] Planet PJ Diaz L Kolokotronis SO Narechania A Reyes J Xing G Parallel epidemics of community-associated methicillin-resistant staphylococcus aureus USA300 infection in North and South America. J Infect Dis. 2015;212(12):1874-82. 10.1093/infdis/jiv320 26048971 PMC4655856

[38] Gwizdala RA Miller M Bhat M Vavagiakis P Henry C Neaigus A Staphylococcus aureus colonization and infection among drug users: identification of hidden networks. Am J Public Health. 2011;101(7):1268-76. 10.2105/AJPH.2010.300028 21653250 PMC3110233

[39] Colombo C Senn G Bürgel A Ruef C Clearance of an epidemic clone of methicillin-resistant Staphylococcus aureus in a drug-use network: a follow-up study in Switzerland. Scand J Infect Dis. 2012;44(9):650-5. 10.3109/00365548.2012.672766 22497490

[40] Kwiatkowska RM Manley P Sims B Lamagni T Ready D Coelho J Outbreak Control Team Outbreak of group A Streptococcus emm94.0 affecting people who inject drugs in southwest England, April 2017. Am J Infect Control. 2018;46(2):238-40. 10.1016/j.ajic.2017.08.011 29031429

[41] Bundle N Bubba L Coelho J Kwiatkowska R Cloke R King S Ongoing outbreak of invasive and non-invasive disease due to group A Streptococcus (GAS) type emm66 among homeless and people who inject drugs in England and Wales, January to December 2016. Euro Surveill. 2017;22(3):30446. 10.2807/1560-7917.ES.2017.22.3.30446 28128090 PMC5322289

[42] Athey TBT Teatero S Sieswerda LE Gubbay JB Marchand-Austin A Li A High Incidence of Invasive Group A Streptococcus Disease Caused by Strains of Uncommon emm Types in Thunder Bay, Ontario, Canada. J Clin Microbiol. 2016;54(1):83-92. 10.1128/JCM.02201-15 26491184 PMC4702752

[43] Septimus EJ Schweizer ML Decolonization in Prevention of Health Care-Associated Infections. Clin Microbiol Rev. 2016;29(2):201-22. 10.1128/CMR.00049-15 26817630 PMC4786886

[44] European Centre for Disease Prevention and Control and European Monitoring Centre for Drugs and Drug Addiction (ECDC and EMCDDA). Prevention and control of infectious diseases among people who inject drugs. Stockholm: ECDC/EMCDDA; 2011. Available from: http://www.emcdda.europa.eu/publications/ecdc-emcdda-guidance_en

[45] Phillips KT Stein MD Risk practices associated with bacterial infections among injection drug users in Denver, Colorado. Am J Drug Alcohol Abuse. 2010;36(2):92-7. 10.3109/00952991003592311 20337504 PMC4869685

[46] Vlahov D Sullivan M Astemborski J Nelson KE Bacterial infections and skin cleaning prior to injection among intravenous drug users. Public Health Rep. 1992;107(5):595-8. 1410243 PMC1403704

[47] Somers CJ Bridgeman J Keenan E Nasal carriage prevalence of meticillin resistant (MRSA) and meticillin sensitive (MSSA) Staphylococcus aureus for subjects attending a Dublin methadone clinic. J Infect. 2010;60(6):494-6. 10.1016/j.jinf.2010.03.012 20346974

[48] Small W Wood E Lloyd-Smith E Tyndall M Kerr T Accessing care for injection-related infections through a medically supervised injecting facility: a qualitative study. Drug Alcohol Depend. 2008;98(1-2):159-62. 10.1016/j.drugalcdep.2008.05.014 18650034

[49] Semaan S Fleming P Worrell C Stolp H Baack B Miller M Potential role of safer injection facilities in reducing HIV and hepatitis C infections and overdose mortality in the United States. Drug Alcohol Depend. 2011;118(2-3):100-10. 10.1016/j.drugalcdep.2011.03.006 21515001

[50] Harris RE Richardson J Frasso R Anderson ED Perceptions about supervised injection facilities among people who inject drugs in Philadelphia. Int J Drug Policy. 2018;52:56-61. 10.1016/j.drugpo.2017.11.005 29241143

[51] Cimolai N MRSA and the environment: implications for comprehensive control measures. Eur J Clin Microbiol Infect Dis. 2008;27(7):481-93. 10.1007/s10096-008-0471-0 18273652

